# Optimizing the sensitivity of detection of respiratory syncytial virus infections in longitudinal studies using the combination of weekly sample testing and biannual serology

**DOI:** 10.1093/aje/kwaf271

**Published:** 2025-12-09

**Authors:** Shannon C Conrey, Daniel C Payne, Maria Deza Leon, Monica Epperson, Melissa M Coughlin, Allison R Burrell, Claire P Mattison, Rachel M Burke, Julia M Baker, Natalie J Thornburg, Meredith L McMorrow, Mary Allen Staat, Ardythe L Morrow

**Affiliations:** Department of Quantitative and Population Health Sciences, Case Western Reserve University School of Medicine, Cleveland, OH, United States; Department of Environmental and Public Health Sciences, University of Cincinnati College of Medicine, Cincinnati, OH, United States; Division of Infectious Disease, Cincinnati Children’s Hospital Medical Center, Cincinnati, OH, United States; Department of Pediatrics, University of Cincinnati College of Medicine, Cincinnati, OH, United States; Department of Infectious Disease, Children’s Mercy Kansas City, Kansas City, MO, United States; National Center for Immunization and Respiratory Diseases, Centers for Disease Control and Prevention, Atlanta, GA, United States; National Center for Immunization and Respiratory Diseases, Centers for Disease Control and Prevention, Atlanta, GA, United States; Division of Infectious Disease, Cincinnati Children’s Hospital Medical Center, Cincinnati, OH, United States; National Center for Immunization and Respiratory Diseases, Centers for Disease Control and Prevention, Atlanta, GA, United States; Cherokee Nation Operational Solutions, Tulsa, OK, United States; Global Development Division, The Gates Foundation, Seattle, WA, United States; National Center for Immunization and Respiratory Diseases, Centers for Disease Control and Prevention, Atlanta, GA, United States; National Center for Immunization and Respiratory Diseases, Centers for Disease Control and Prevention, Atlanta, GA, United States; National Center for Immunization and Respiratory Diseases, Centers for Disease Control and Prevention, Atlanta, GA, United States; Division of Infectious Disease, Cincinnati Children’s Hospital Medical Center, Cincinnati, OH, United States; Department of Pediatrics, University of Cincinnati College of Medicine, Cincinnati, OH, United States; Department of Environmental and Public Health Sciences, University of Cincinnati College of Medicine, Cincinnati, OH, United States; Department of Pediatrics, University of Cincinnati College of Medicine, Cincinnati, OH, United States

**Keywords:** cohort studies, decision trees, incidence rate, infectious disease epidemiology, misclassification bias, selection bias, attrition bias, epidemiologic methods

## Abstract

Cohort studies are often challenged by incomplete adherence to sampling regimens, limiting the full capture of disease burden. We describe the detection of respiratory syncytial virus (RSV) infections achieved in a birth cohort using a combination of weekly nasal sample testing and serology. The Pediatric Respiratory and Enteric Viral Acquisition and Immunogenesis Longitudinal Cohort followed 245 maternal–child dyads from birth to age 18 to 24 months. Weekly mid-turbinate nasal swabs were tested for RSV using real-time polymerase chain reaction (RT-qPCR). Serum was tested for RSV pre-fusion F IgG and IgA antibody at age 6 weeks and biannually from 6 to 24 months. Mixed effects classification and regression trees identified antibody thresholds consistent with a RT-qPCR-identified RSV infection using a subset of participants having ≥90% weekly sample adherence (*n* = 53, 21%). Resulting thresholds were applied to participants with either ≥70% of weekly samples or serum at age 18 to 24 months (*n* = 194, 79%). Incidence rates were compared using Fisher's exact test. Classification and regression trees identified a log_10_ change in IgG > 0.32 or IgA > 0.20 as indicative of an RSV infection. Comparing RT-qPCR-only to a combination of RT-qPCR and serology, RSV cumulative incidence (49% vs 75%, *P* < .001) and incidence rate (0.33 vs 0.71 infections/child-year, *P* < .001) increased; these rates did not differ from those calculated in those with ≥90% sample adherence.

## Teaser

**Key messages**
 

- We sought to develop a method to maximize RSV infection detection to optimize estimation of disease burden in longitudinal studies, which are prone to incomplete protocol adherence to weekly sample collection.
- Using a combination of weekly sample submissions and regular IgG and IgA serology, we identified incident RSV infections in participants with lower weekly sample submissions.
- The combination of weekly samples and periodic serology can be used to increase power, reduce selection bias, and improve study compatibility in infectious disease cohort studies.

## Introduction

Cohort studies can provide powerful insights into the natural history of acute infections through longitudinal testing and follow-up.^1^^,^^2^ However, complete ascertainment of infections depends on participant adherence to frequent sample collection, which is highly variable.^1^^,^^3^^-^^5^ Restricting analyses to participants with high sample adherence optimizes infection detection,^3^^,^^4^^,^^6^ but reduces sample size and may differentially exclude participants, resulting in potential selection bias.^1^^,^^7^ Reducing participant burden by collecting samples only when symptomatic may increase participation but limits the ability to detect asymptomatic infections, underestimating incidence.^8^^,^^9^

Serological assays in cohort studies provide another approach to detecting infections and are less reliant on frequent sampling. However, most serologic assays are validated to identify seropositivity, defined as having ever been infected, requiring further evaluation to detect repeat infections and calculate incidence rates.^10^ Furthermore, serological detections alone cannot date an infection, and therefore restrict the ability to assess symptomatology, healthcare utilization, or time-varying factors associated with disease.^8^

The lack of consistent methods to determine respiratory syncytial virus (RSV) incidence results in heterogeneity and lack of comparability across studies.^8^^,^^11^^-^^14^ Furthermore, identifying mild and asymptomatic infections is essential to understanding the natural history of immune development, necessary to assess and optimize vaccine effectiveness.^9^^,^^15^ Here, we describe an integrated method to identify incident RSV infections using a combination of real-time polymerase chain reaction (RT-qPCR)-tested nasal swabs and biannual serologic collection in a 2-year birth cohort. We compare detection sensitivity by methodologic decision points and evaluate the differences by methods in RSV incidence, statistical power, and selection and misclassification biases.

## Methods

### Study cohort

The Pediatric Respiratory and Enteric Viral Acquisition and Immunogenesis Longitudinal (PREVAIL) Cohort is a 2-year birth cohort conducted in Greater Cincinnati, Ohio from April 2017 to July 2020. Institutional review board approvals were obtained from the US Centers for Disease Control and Prevention, Cincinnati Children's Hospital Medical Center, and enrolling birth hospitals. Full study methods have been published.^2^ Briefly, prenatal enrollment included mothers who were ≥34 weeks gestation, ≥18 years of age, and expecting a healthy, singleton child. Children were followed from birth until age 2 years, including weekly symptom surveillance via text message, weekly nasal swab and stool sample submission, and regular in-person study visits and blood draws.

### RSV detection

Caregivers collected mid-turbinate nasal samples each week using flocked swabs (Copan Diagnostics, Inc.), placed into vials containing BD Universal Viral Transport medium (Becton, Dickinson, and Co., Franklin Lakes, NJ) and delivered by courier to the study laboratory within 48 hours of collection. Swabs were tested for respiratory pathogens, including RSV A and B, using the Luminex multiplex RT-qPCR NxTAG Respiratory Pathogen Panel assay.^16^ Detections were considered part of a continuous RSV infection if the same subtype was detected ≤ 30 days apart.^8^ RT-qPCR-detected infections were dated using the date of the first RT-qPCR–positive.

Serum samples were collected using a standardized protocol at birth and at in-clinic study visits at ages 6 weeks and 6, 12, 18, and 24 months. RSV pre-fusion F IgA and IgG antibodies were measured using the MesoScale Discovery Respiratory Panel 1 multiplex serology assay (MesoScale Diagnostics, Rockville MD) according to the manufacturer's instructions, calculated in arbitrary units (AUs) per mL using standard curves with manufacturer provided calibrators. Concentrations below or above the level of quantification (LOQ) were imputed as half the lower LOQ or the upper LOQ, respectively.

### Definition of adherence levels

**Figure 1:**
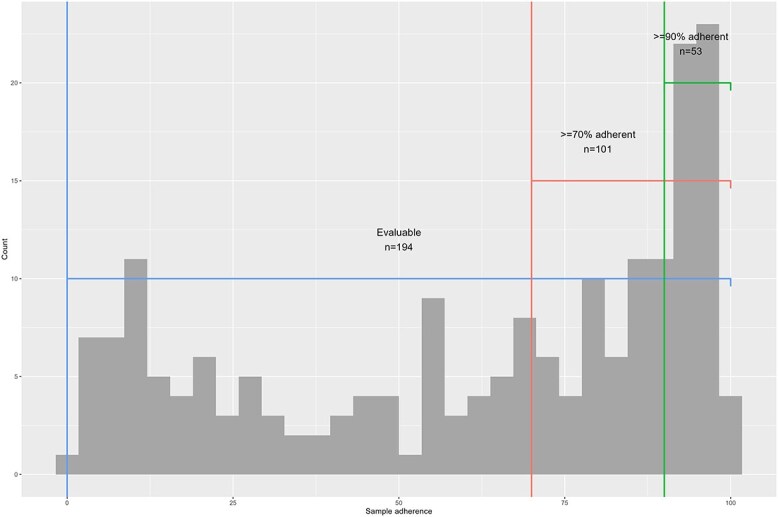
Distribution of weekly sample adherence by subset inclusion criteria. Participants in the PREVAIL cohort were considered evaluable for infectious disease outcomes if completed ≥18 months of the study and submitted either ≥70% of weekly samples or provided a serum sample ≥18 months of age. To reduce misclassification bias in studies requiring more consistent sample submission, analysis was restricted to evaluable participants who were ≥70% weekly sample adherent. When identifying training data for predictive modeling, participants who were ≥90% weekly sample adherent were included, reducing time between missed samples. Abbreviation: PREVAIL, Pediatric Respiratory and Enteric Viral Acquisition and Immunogenesis Longitudinal Cohort.

To replicate study populations for different analytic designs, we identified subsets using varying weekly sample adherence levels as inclusion criteria. It is important to note that each of these rules is applied to the prior subset, so the subsets are nested, and adherence level represents proportion of weekly samples submitted over the course of the entire study (Figure 1). Participants were considered evaluable for infectious outcomes if they participated in the study ≥18 months and submitted either ≥70% of weekly samples or provided a serum sample at ≥18 months of age. For studies reliant on RT-qPCR only, we subsetted evaluable participants who were ≥70% weekly sample adherent. For analysis requiring minimal gaps in submissions, we subsetted evaluable participants who submitted ≥90% of weekly samples.

### Identification of thresholds

Using the ≥90% adherent subset, we applied a mixed-effects Classification and Regression Tree (CART) analysis to generate detection thresholds in serum antibody consistent with having an incident RSV infection during the interim between blood draws (study interval). CART models consider all possible combinations and levels of variables to recursively split data, selecting thresholds in the available predictors that result in the most accurate terminal nodes.^17^ Study intervals were identified as positive or not positive for RSV based on the presence of a RT-qPCR–identified RSV infection within the study interval. Due to the time required to mount a detectable antibody response after an infection, study intervals with a RT-qPCR positive within 4 weeks of the blood draw were identified as positive during the subsequent study interval. Using the change in antibody levels between blood draws, three possible predictors were included in the CART models: (1) change in log_10_ concentration, (2) fold-change in concentration, and (3) a binary indicator of ≥4-fold change in concentration. A fourth predictor, log_10_ concentration at the time of the blood draw, was also included. CART models for each isotype were constructed without restriction to the number of splits or application of variable weights, first for each predictor separately to identify method-specific thresholds, then in combination to identify the most accurate method.

After applying the selected method's thresholds, serologic detections associated with a positive study interval were considered confirmatory of the RT-qPCR-positive, while serologic detections in a not-positive study interval were considered new incident infections. Investigations, including timing of the serum sample relative to the RT-qPCR positive, weekly sample adherence, and reported respiratory symptoms during the study interval, were conducted for all unconfirmed RT-qPCR–identified infections and new incident infections. Once detection thresholds were established in ≥90% adherent participants, they were applied to all evaluable participants.

### Statistical analysis

Fisher's exact test or Kruskal-Wallis were used to compare sociodemographic factors among the adherence-defined subsets. Cumulative incidence in each subset was calculated for infections detected using RT-qPCR only and by combining RT-qPCR and serology. To calculate incidence rates, we calculated weeks-at-risk by multiplying the number of infections by 4 weeks, as RSV detections within 30 days were considered part of a continuous infection, then subtracting the resulting number from the total weeks of follow-up. Incidence rates were reported per child year. Fisher's exact test with Holms corrections was used to compare proportions of infections identified by RT-qPCR and by serology, as well as cumulative incidence and incidence rate for each subset.

To evaluate the effect of study design criteria on power, we calculated detectable effect size at 80% power for a χ^2^ test for each of our three adherence subsets. To examine the impact on misclassification and selection biases, we compared the demographic factors between those RSV-infected vs not RSV-infected in our evaluable subset using the RT-qPCR–only method, serology-only method, and the combined method using Kruskal-Wallis for continuous and Fisher's exact test for categorical data. All analyses were conducted using the R Environment for Statistical Computing (version 4.2.3).

## Results

**Figure 2:**
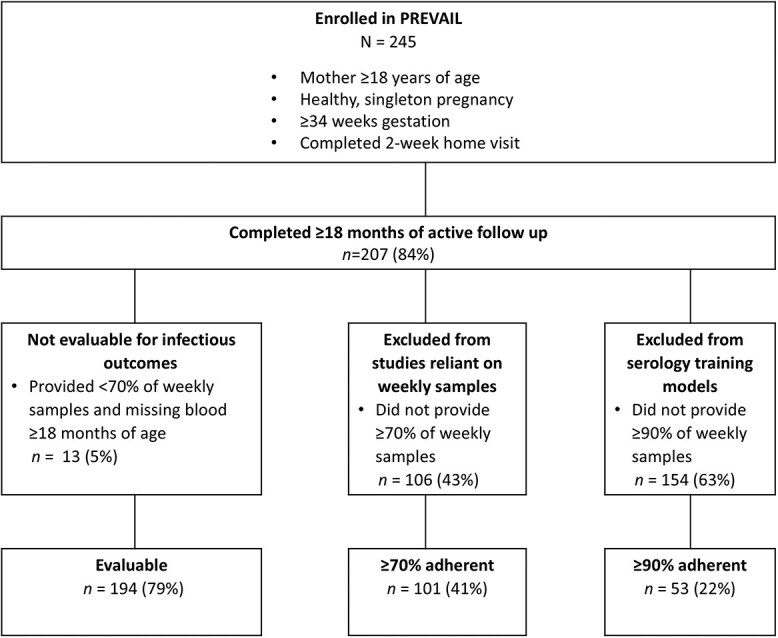
STROBE flowchart, indicating the inclusion and exclusion criteria for each subset. Abbreviation: STROBE, Strengthening the Reporting in Observational Studies in Epidemiology.

PREVAIL Cohort enrolled 245 participants; 194 (79%) were defined as evaluable, 101 (41%) were ≥70% adherent, and 53 (22%) were ≥90% adherent (Figure 2). Each increase in adherence criteria resulted in a smaller subset with greater proportions of subjects who identified as White race, higher income, privately insured, higher education level, and who breastfed for longer durations (Table 1). Nearly half of evaluable participants were included due to providing a blood sample at 18 or 24 months. These participants were more likely to identify as Black race, lower education level, publicly insured and family income <$50 000/year compared to those with higher weekly sample adherence. The evaluable subset was demographically similar to the Cincinnati metropolitan area.^18^

**Table 1:** Demographics by subset inclusion criteria.

			**Evaluable^**a**^*, N* = 194**			
				**≥70% Adherent^**b**^*, N* = 101**		
	**Enrolled*, N* = 245** ^**c**^	**Non-evaluable/excluded from analysis, *n* = 51** ^**c**^	**<70% weekly samples, but serum ≥18 M*, n* = 93** ^**c**^	**70% ≤ weekly, samples < 90%*, n* = 48** ^**c**^	**≥90% weekly samples, *n* = 53** ^**c**^	***P***
Nasal swab adherence	61 (22, 88)	29 (13, 54)	29 (11, 51)	80 (74, 87)	94 (91, 95)	<.001
Maternal age	29.6 (25.7, 33.2)	26.1 (23.2, 30.3)	28.7 (25.2, 31.3)	31.6 (28.3, 35.0)	33.0 (30.0, 34.6)	<.001
Race						<.001
Black	107 (44%)	25 (49%)	65 (70%)	11 (23%)	6 (11%)	
White/other	138 (56%)	26 (51%)	28 (30%)	37 (77%)	47 (89%)	
Hispanic ethnicity	6 (2.4%)	0 (0%)	1 (1.1%)	2 (4.2%)	3 (6%)	.2
Insurance type						<.001
Private	106 (43%)	13 (25%)	15 (16%)	30 (63%)	48 (91%)	
Public	139 (57%)	38 (75%)	78 (84%)	18 (38%)	5 (9.4%)	
Marital status						<.001
Married/partner	163 (67%)	29 (57%)	47 (51%)	40 (83%)	47 (89%)	
Single	82 (33%)	22 (43%)	46 (49%)	8 (17%)	6 (11%)	
Maternal education						<.001
High school or less	115 (47%)	34 (67%)	63 (68%)	13 (27%)	5 (9.4%)	
≥2+ years post-secondary	130 (53%)	17 (33%)	30 (32%)	35 (73%)	48 (91%)	
Family income						<.001
<$25 000	88 (36%)	27 (53%)	49 (53%)	10 (21%)	2 (3.8%)	
$25 000-$50 000	49 (20%)	13 (25%)	26 (28%)	7 (15%)	3 (5.7%)	
>$50 000	108 (44%)	11 (22%)	18 (19%)	31 (65%)	48 (31%)	
Infant sex						.2
Female	127 (52%)	31 (61%)	50 (54%)	24 (50%)	22 (42%)	
Male	118 (48%)	20 (39%)	43 (46%)	24 (50%)	31 (58%)	
Delivery mode						.14
C section	94 (38%)	16 (31%)	30 (32%)	23 (48%)	25 (47%)	
Vaginal	151 (62%)	35 (69%)	63 (68%)	25 (52%)	28 (53%)	
Birthweight (kg)	3.29 (2.95, 3.53)	3.27 (2.87, 3.46)	3.22 (2.83, 3.50)	3.32 (3.09, 3.62)	3.37 (3.07, 3.61)	.2
Initiated breastfeeding	212 (87%)	41 (80%)	75 (81%)	45 (94%)	51 (96%)	.023
Days of breastfeeding	83 (16, 312)	46 (9, 138)	33 (2, 123)	227 (47, 430)	310 (122, 428)	<.001

Abbreviation: PREVAIL, Pediatric Respiratory and Enteric Viral Acquisition and Immunogenesis Longitudinal Cohort.

^a^Participants in the evaluable subset (*n* = 194) participated in PREVAIL at least 18 months and contributed a serum sample at ≥18 months of age or ≥70% of weekly samples.

^b^Highly adherent participants were defined as evaluable participants with either ≥70% (*n* = 101) or ≥90% (*n* = 53) weekly sample adherence.

^c^Median (Q1, Q3); *n* (%).

### Identification of RSV thresholds in the ≥90% adherent participants

**Figure 3:**
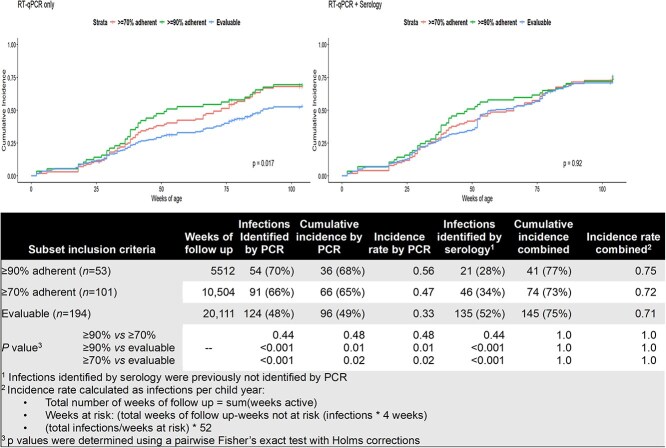
Comparison of incidence by adherence level and detection method. Cumulative incidence comparisons by subset adherence level demonstrating the attenuation of differences between adherence levels in cumulative incidence and incidence density when using a combination of RT-qPCR and serological RSV detections. *P* values in the plots represent differences by log-likelihood. Abbreviations: RSV, respiratory syncytial virus; RT-qPCR, real-time polymerase chain reaction.

RT-qPCR identified 54 RSV infections (36 first, 18 repeat infections) occurring in 68% of children (*n* = 36) with an incidence rate of 0.56 infections/child-year (Figure 3). While the CART-identified thresholds (Table S1) for the three predictors related to change in antibody had high percent agreement with RT-qPCR, the threshold selected for log_10_ concentration resulted in a high number of serial positives, overestimating incident infections. Seropositivity comparisons with the validated ROC curves were determined using IgA only, as all children were seropositive by IgG between birth and 6 months of age. All methods had high percent agreement with the validated ROC curves in terms of detecting seropositivity.

**Figure 4:**
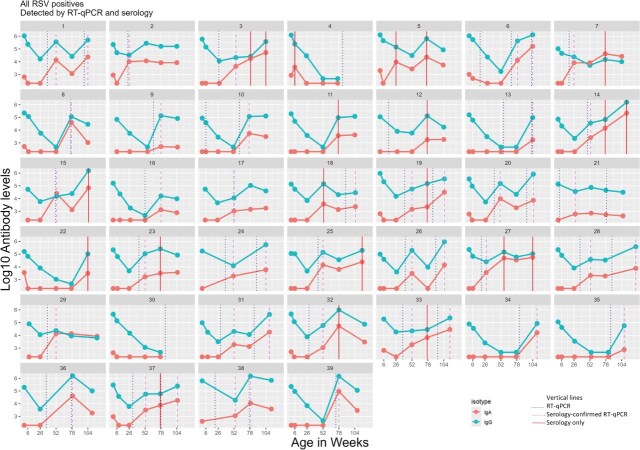
RSV detections in highly adherent PREVAIL children. Line graphs of IgA (coral) and IgG (aqua) antibodies at each study interval, with vertical lines indicating RT-qPCR-identified infections (dotted line), serology confirmation of the RT-qPCR-identified infections (dashed line), and serology-only identified infections (solid line). Participants were ≥90% weekly sample adherent over the course of the 2-year PREVAIL cohort. Abbreviations: PREVAIL, Pediatric Respiratory and Enteric Viral Acquisition and Immunogenesis Longitudinal Cohort; RSV, respiratory syncytial virus; RT-qPCR, real-time polymerase chain reaction.

In the combined model, CART identified a single variable and threshold per isotype, log_10_ increases of >0.202 AU or >0.32 AU, for IgA and IgG, respectively, from the previous blood draw as the most accurate predictor of an incident RSV infection, hereafter referred to concentration change. Using the concentration change thresholds, we identified 21 previously undetected RSV infections in 17 children, including 4 infections in 3 children without a detectable RT-qPCR-identified infection (Figure 4, participants 5, 11, 22). Most (*n* = 19, 90%) of these new infections were identified using IgA; 33% (*n* = 7) were identified using IgA only, including both new infections in the first 6 months of life. Nearly half of new infections (*n* = 10, 48%) were detected following a study interval with very high weekly sample adherence (mean 98.0% ±2.8), while 52% (*n* = 11) were detected following study intervals of lower adherence (mean, 64.7%; ±14.6). Nearly all children (15/17, 88%) reported respiratory symptoms by text survey during the study interval prior to the serologic positive.

Our thresholds confirmed 47/54 (87%) RT-qPCR–identified infections. Of the seven unconfirmed (Figure 4), two children did not have a blood draw after the RT-qPCR–positive (participants 4 and 30) and five children (participants 6, 10, 12, 13, 21) did not report any respiratory symptoms during the interval. However, given the high specificity of the Luminex NxTAG RPP (1; 95% CI, 0.97-1), these infections were retained for incidence calculations. After combining RT-qPCR- and concentration change-identified infections, we identified 77 infections in 39 children, resulting in 77% cumulative incidence and 0.75 infections/child-year (Figure 3).

### Application to all evaluable participants

RT-qPCR detected 124 RSV infections (96 first, 28 repeat infections) in 96/194 (49%) children, with an incidence rate of 0.33 infections/child-year (Figure 3). Concentration change identified 133 new infections, including 85 infections in 49 children who previously did not have an RT-qPCR–detected infection (Table 2); 51/133 (38%) were identified using IgA only, 3/133 (2%) were identified using IgG only, and 79/133 (60%) met both criteria. IgA only identified 85% (11/13) of new infections in the first year of life and 39% (30/76) of repeat infections. A comparison of results by each detection method can be found in Table S2.

**Table 2:** New serologic RSV detections by detection method in 194 PREVAIL children.

			**Method used to identify infection**		
		**All new concentration change-identified infections, *n* = 133**	**IgA only, *n* = 51 (38%)**	**IgG only, *n* = 3 (2%)**	**Both, *n* = 79 (60%)**
Age category	<6 months 6-11 months 12-17 months 18-24 months	2 (2%) 11 (8%) 59 (44%) 61 (46%)	2 (4%) 9 (18%) 19 (37%) 21 (41%)	0 (0%) 0 (0%) 2 (67%) 1 (33%)	0 (0%) 2 (3%) 38 (48%) 39 (49%)
Infection number	First Second Third	57 (43%) 61 (46%) 15 (11%)	21 (41%) 28 (55%) 2 (4%)	0 (0%) 2 (66%) 1 (33%)	36 (46%) 31 (39%) 12 (15%)

Abbreviations: CART, Classification and Regression Tree; PREVAIL, Pediatric Respiratory and Enteric Viral Acquisition and Immunogenesis Longitudinal Cohort; RSV, respiratory syncytial virus.Mixed effects CART analysis was used to identify thresholds of positivity using pre-fusion F IgA and IgG assays from serum collected at 6 weeks and 6, 12, 18, and 24 months of age. CART-derived thresholds of >2.02 change in log_10_ concentration of IgA or a >0.32 change in log_10_ concentration of IgG were applied to PREVAIL participants who participated in the study at least 18 months and were either ≥70% adherent in weekly sample submission or provided a serum sample at 18 or 24 months of age.

Of the 124 RT-qPCR–identified infections, 109 (88%) were confirmed serologically; nearly half (7/15, 47%) of the unconfirmed RT-qPCR infections occurred in the first 6 months of life and 53% (8/15) did not report symptoms. When combining RT-qPCR and serology, 259 infections were identified in 145 children, increasing cumulative incidence to 75% by 2 years of age (*P* < .001), and incidence rate to 0.71 infections/child-year (*P* < .001).

### Comparison of incidence, power achieved, and bias by protocol adherence and method

Using RT-qPCR–identified infections, cumulative incidence at 2 years of age (68%, 65%, 49%, *P* = .02) and incidence rate (0.56, 0.47, 0.33, *P* = .003) varied among the ≥90% adherent, ≥70% adherent, and the evaluable group, respectively. (Figure 3). Using our integrated approach, no differences remained in cumulative incidence (77%, 73%, 75%, *P* = .68) or incidence rate (0.75, 0.72, 0.71, *P* = .20).

**Figure 5:**
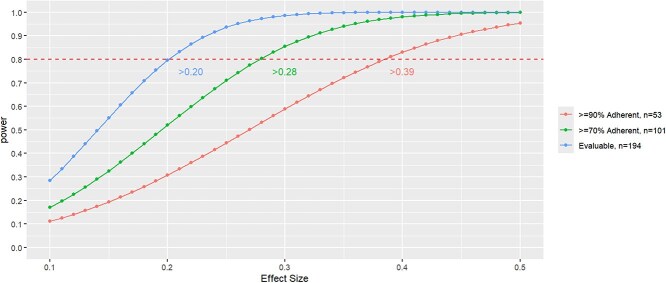
Power achieved by effect size for each of the adherence criteria. The dotted red horizontal line represents the point at which 80% power is achieved. The figure labels represent the effect size detectable at 80% power for each adherence level.

In comparing power using χ^2^ tests by subset (Figure 5), ≥80% power was achieved with an effect size ≥39% for the ≥90% adherent, ≥28% for the ≥70% adherent, and >20% for all eligible participants.

When we compared those who did and did not have an RT-qPCR–identified RSV infection (Table 3), differences by race, insurance type, marital status, maternal education, and family income were detected. However, after adding serologically identified infections, no differences remained in these factors.

**Table 3:** Comparison of RSV infections by demographics using RT-qPCR-only and a combination of RT-qPCR- and serology-identified infections among all evaluable participants.

**Variables**		**Evaluable*,*** ***N* = 194**^**a**^	**Positive by RT-qPCR, *n* = 96 (49%)** ^**a**^	***P*** ^***b***^	**Positive by Serology, *n* = 143 (74%)** ^**a**^	***P*** ^***b***^	**Positive Combined, *n* = 145 (75%)** ^**a**^	***P*** ^***b***^
Maternal age		30.2 (26.9, 33.7)	30.6 (28.4, 34.3)	.06	30.2 (27.0, 33.7)	.9	30.3 (27.2, 33.7)	.8
Race	Black	82 (42%)	27 (28%)	**<.001**	60 (42%)	.9	60 (41%)	.5
	White/other	112 (58%)	69 (72%)		83 (58%)		85 (59%)	
Hispanic	Yes	6 (3%)	4 (4%)	.4	5 (4%)	>.9	5 (3%)	>.9
	No	188 (97%)	92 (96%)		138 (96%)		140 (97%)	
Insurance	Private	93 (48%)	59 (61%)	**<.001**	65 (45%)	.2	67 (46%)	.4
	Public	101 (52%)	37 (39%)		78 (55%)		78 (54%)	
Marital status	Married/partner	134 (69%)	75 (78%)	**.007**	99 (69%)	>.9	101 (70%)	.8
	Single	60 (31%)	21 (22%)		44 (31%)		44 (30%)	
Maternal education	High school or less	81 (42%)	24 (25%)	**<.001**	58 (41%)	.6	58 (40%)	.4
	≥2 years post-secondary	113 (58%)	72 (75%)		85 (59%)		87 (60%)	
Family income	<$25 000	61 (31%)	19 (20%)	**<.001**	43 (30%)	.5	44 (30%)	.4
	$25 000-$50 000	36 (19%)	13 (14%)		32 (22%)		30 (21%)	
	>$50 000	97 (50%)	64 (67%)		68 (48%)		71 (49%)	
Infant sex	Female	96 (49%)	46 (48%)	.7	76 (53%)	.3	76 (52%)	.2
	Male	98 (51%)	50 (52%)		67 (47%)		69 (48%)	

Abbreviation: RT-qPCR, real-time polymerase chain reaction.

^a^Median (Q1, Q3); *n* (%).

^b^Compared to all evaluable. Bolded values represent p<0.05.

Using RT-qPCR–only, 49/194 (25%) of evaluable children were misclassified as never having an RSV infection by age 2 years. Furthermore, 25 RT-qPCR-identified infections were misclassified as first infections; 19 of these were reclassified as second and 6 were reclassified as third after adding concentration change-identified infections.

### Sensitivity analysis

Unconfirmed RT-qPCR–positives were removed from incidence rate calculations for each adherence subset (≥90% adherent, ≥70% adherent, evaluable). The adjusted incidence rates (0.71, 0.65, 0.66, respectively) did not differ from each other (*P* = .20) or from the previous estimates (*P* = .22).

## Discussion

The PREVAIL Cohort accurately and more completely identified RSV infections by age 2 years by integrating RT-qPCR–tested nasal swabs with biannual blood draws assayed for both RSV IgG and IgA antibodies. We demonstrate that this combined method resulted in more robust RSV incidence estimates while simultaneously increasing power and reducing selection and misclassification biases. In addition, this method allows comparisons across detection methods, including those that rely on RT-qPCR–only and serology-only procedures to detect RSV infections.

Our reported cumulative incidence and incidence rates (Figure 3) are consistent with findings from recent international cohorts employing a range of methods. The Australian ORChID Cohort reported an RSV incidence rate of 0.46 (95% CI, 0.37-0.58) infections/child-year using weekly RT-qPCR detections from birth to age 2 years.^14^ Nyiro et al reported RSV seropositivity of 83% by age 2 years in all children admitted to a rural Kenyan hospital using crude ELISA assays,^13^ while Kutsaya et al reported 68% seropositivity by age 2 years in healthy Finnish children by IgG enzyme immunoassay.^12^ While the heterogeneity of methods across these studies makes direct comparisons difficult, our findings are consistent with each, underscoring the value of our integrated approach to detections.

The transplacental transfer of IgG combined with its durability leads to challenges in identifying early-life and repeat RSV infections when only IgG is measured.^8^^,^^12^^,^^13^ For example, Kutsaya et al detected no discernable rise in IgG in five symptomatic, PCR-confirmed RSV infections occurring prior to 9 months of age and concluded that they underestimated incidence rates due to the attenuated rise in IgG associated with repeat infection.^12^ Nyiro et al reported that 100% of study children were seropositive by IgG at 1 month of age and estimated the average duration of maternally acquired antibodies to be 4.7 months.^13^ Our use of CART to identify thresholds in IgA and IgG rather than a standard 4-fold rise in IgG better enabled detection of these early and repeat infections; 98% of all serologically detected infections were detected using IgA, with 38% (*n* = 51) using IgA only including 85% of new infections between birth-12 months, 33% of new infections in the second year of life and 39% of repeat infections (Table 2). Removing these infections would result in a significantly decreased incidence rate of 0.56 infections/child year (*P* = .01) in the evaluable group. We should note that our blood collections were twice-yearly; detection of IgA F antigen declines over time,^19^ making the frequency of blood sampling an important decision point in assay selection.

Combining RT-qPCR- and serology-detected infections eliminated differences in cumulative incidence and incidence rates by adherence-defined subsets, allowing for the inclusion of nearly 80% of enrolled participants and increasing power. In addition, the reduction in selection bias associated with sociodemographic factors using our integrated approach may provide a more robust and generalizable estimation of disease burden.^1^^,^^3^^,^^5^^,^^6^ Finally, the reduction in misclassifications, particularly in order of infection, is instrumental for understanding the natural history of immune response, necessary to estimate and optimize vaccine effectiveness.^9^

It is important to note that many caregivers may hesitate to participate in a pediatric study with regular blood draws, which could result in volunteer bias at enrollment.^20^ PREVAIL screened and approached 1206 mothers in the third trimester of pregnancy for potential enrollment; 941 (78%) met eligibility but declined to enroll.^2^ Those who consented to enroll in the study did not differ racially from those who declined.^2^ Most of those who declined did not provide a specific reason (*n* = 843, 90%), but it is probable that some were hesitant about the blood draws. However, only 14% (*n* = 14) who provided a reason cited the blood draws; most (*n* = 56, 57%) had delivered prior to contact and 29% (*n* = 28) cited the time commitment. Finally, participants who met our evaluation criteria by providing a blood sample improved the representativeness of our study (Table 1). We concluded that blood draws were not a significant source of bias in our study enrollment.

Although incidence calculations using serology-only were nearly identical to those combining RT-qPCR and serology, serologically identified infections cannot be precisely dated, hindering examinations of severity or symptomatology of infections. Such examinations are important for RSV, as reinfections are common throughout life,^12^^,^^13^^,^^21^^,^^22^ with rapidly waning immunity following exposure. Effectiveness studies of two RSV immunization strategies report strong protection against severe illness within the first 6 months of receipt, followed by waning protection.^23^^-^^25^ Gauging effectiveness of immunization to prevent or reduce severity of symptoms requires precise dating of the immunization event, infection, and symptom onset, while accurate incidence calculations and characterization of the natural history of immune development requires identifying all infections, including those that are mild or asymptomatic. Our integrated method provides data for both purposes.

Our study is not without limitations. While the ≥90% adherent group rarely skipped submissions in consecutive weeks, a weekly sample protocol can miss short, transient infections. Furthermore, weekly sample adherence was calculated over the study duration, but some participants experienced periods of lower adherence, including half of those with serologically identified new infections. As these participants provided the training data for the CART models, missed infections may have resulted in a misclassification of study intervals as “not positive” and thereby reduced the sensitivity of the detections. It is also possible that many serology-only detected infections, especially in the high adherence group, represent transient exposures that provided a boosting effect rather than true RSV infections. However, the presence of respiratory symptoms occurring in 88% of the intervals with a new detection and the consistency of our incidence calculations with other studies^12^^-^^14^ supports our inclusion of these infections in our calculations.

Our study also has several important strengths. Using CART to identify thresholds using highly adherent children rather than relying on a standardized 4-fold rise increased our detections, while applying both IgA and IgG thresholds allowed for the detection of early and repeat infections. While our sample size was modest, our method allowed us to maximize power by including participants with lower weekly sample adherence. The identification of serology-only infections during periods of high sample adherence suggests that this method may also address detection sensitivity deficits due to the use of a mid-turbinate nasal swab or sample quality due to parental collection. Finally, our method reduced potential sociodemographic biases associated with limiting analysis to highly adherent participants, resulting in greater study robustness and power.

## Conclusions

Using CART analysis to identify serologic thresholds consistent with RSV infection integrated with weekly RT-qPCR-detections allowed the full capture of RSV infections, facilitated improved accuracy of disease burden estimates, and provided a better understanding of RSV immunologic events in infancy and early childhood. While larger studies are needed to establish generalizable serology thresholds for RSV infection, use of IgG and IgA serology combined with weekly nasal swab testing is a way for cohort studies to maximize power and reduce biases in RSV detections.

## Supplementary Material

Web_Material_kwaf271

## Data Availability

Data and statistical code available upon approved emailed request. This manuscript was available in preprint: https://www.medrxiv.org/content/10.1101/2025.09.18.25336083v1.

## References

[1] Howe LD, Tilling K, Galobardes B, et al. Loss to follow-up in cohort studies: bias in estimates of socioeconomic inequalities. *Epidemiology.* 2013;24(1):1-9. 10.1097/EDE.0b013e31827623b1 23211345 PMC5102324

[2] Morrow AL, Staat MA, DeFranco EA, et al. Pediatric respiratory and enteric virus acquisition and immunogenesis in US mothers and children aged 0-2: PREVAIL cohort study. *JMIR Res Protoc*. 2021;10(2):e22222. 10.2196/22222 33576746 PMC7910118

[3] Accorsi EK, Qiu X, Rumpler E, et al. How to detect and reduce potential sources of biases in studies of SARS-CoV-2 and COVID-19. *Eur J Epidemiol*. 2021;36(2):179-196. 10.1007/s10654-021-00727-7 33634345 PMC7906244

[4] Teague S, Youssef GJ, Macdonald JA, et al. Retention strategies in longitudinal cohort studies: a systematic review and meta-analysis. *BMC Med Res Methodol*. 2018;18(1):151. 10.1186/s12874-018-0586-7 30477443 PMC6258319

[5] Agampodi S, Tadesse BT, Sahastrabuddhe S, et al. Biases in COVID-19 vaccine effectiveness studies using cohort design. *Front Med (Lausanne)*. 2024;11:1474045. 10.3389/fmed.2024.1474045 39540039 PMC11557388

[6] Stockwell T, Zhao J, Clay J, et al. Why do only some cohort studies find health benefits from low-volume alcohol use? A systematic review and meta-analysis of study characteristics that may bias mortality risk estimates. *J Stud Alcohol Drugs*. 2024;85(4):441-452. 10.15288/jsad.23-00283 38289182

[7] Hellfritzsch M, Pottegård A, Haastrup SB, et al. Cohort selection in register-based studies of direct oral anticoagulant users with atrial fibrillation: an inevitable trade-off between selection bias and misclassification. *Basic Clin Pharmacol Toxicol*. 2020;127(1):3-5. 10.1111/bcpt.13423 32364263

[8] Teoh Z, Conrey S, McNeal M, et al. Burden of respiratory viruses in children less than 2 years old in a community-based longitudinal US birth cohort. *Clin Infect Dis*. 2023;77(6):901-909. 10.1093/cid/ciad289 37157868 PMC10838707

[9] Aguiar M, Van-Dierdonck JB, Mar J, et al. The role of mild and asymptomatic infections on COVID-19 vaccines performance: a modeling study. *J Adv Res*. 2022;39:157-166. 10.1016/j.jare.2021.10.012 35777906 PMC8592646

[10] MedlinePlus . Antibody Serology Tests. National Library of Medicine. Updated September 30, 2024. Accessed February 20, 2025. https://medlineplus.gov/lab-tests/antibody-serology-tests/#:~:text=Antibody%20serology%20tests%20are%20not,past%20infection%20or%20a%20vaccination

[11] Glezen WP, Taber LH, Frank AL, et al. Risk of primary infection and reinfection with respiratory syncytial virus. *Am J Dis Child*. 1986;140(6):543-546. 10.1001/archpedi.1986.02140200053026 3706232

[12] Kutsaya A, Teros-Jaakkola T, Kakkola L, et al. Prospective clinical and serological follow-up in early childhood reveals a high rate of subclinical RSV infection and a relatively high reinfection rate within the first 3 years of life. *Epidemiol Infect*. 2016;144(8):1622-1633. 10.1017/s0950268815003143 26732801 PMC9150639

[13] Nyiro JU, Kombe IK, Sande CJ, et al. Defining the vaccination window for respiratory syncytial virus (RSV) using age-seroprevalence data for children in Kilifi, Kenya. *PloS One*. 2017;12(5):e0177803. 10.1371/journal.pone.0177803 28531224 PMC5439681

[14] Takashima MD, Grimwood K, Sly PD, et al. Epidemiology of respiratory syncytial virus in a community birth cohort of infants in the first 2 years of life. *Eur J Pediatr*. 2021;180(7):2125-2135. 10.1007/s00431-021-03998-0 33634335

[15] Velázquez FR, Matson DO, Calva JJ, et al. Rotavirus infection in infants as protection against subsequent infections. *N Engl J Med*. 1996;335(14):1022-1028. 10.1056/NEJM199610033351404 8793926

[16] Tang Y-W, Gonsalves S, Sun JY, et al. Clinical evaluation of the Luminex NxTAG respiratory pathogen panel. *J Clin Microbiol*. 2016;54(7):1912-1914. 10.1128/JCM.00482-16 27122378 PMC4922127

[17] Fokkema M, Smits N, Zeileis A, et al. Detecting treatment-subgroup interactions in clustered data with generalized linear mixed-effects model trees. *Behav Res Methods*. 2018;50(5):2016-2034. 10.3758/s13428-017-0971-x 29071652

[18] U.S. Census Bureau . *Quick Facts*, Hamilton County, OH. Accessed Web Page, 2025. https://www.census.gov/quickfacts/hamiltoncountyohio

[19] Sankaranarayanan R, Ha B, Sun H, et al. Evaluation of immunoglobulin A enzyme immunoassays to detect primary respiratory syncytial virus infection in infants and young children. *J Infect Dis*. 2025;231(4):1060-1068. 10.1093/infdis/jiae514 39575588 PMC12168064

[20] Jordan S, Watkins A, Storey M, et al. Volunteer bias in recruitment, retention, and blood sample donation in a randomised controlled trial involving mothers and their children at six months and two years: a longitudinal analysis. *PloS One*. 2013;8(7):e67912. 10.1371/journal.pone.0067912 23874465 PMC3706448

[21] Lambert L, Sagfors AM, Openshaw PJ, et al. Immunity to RSV in early-life. *Front Immunol*. 2014;5:466. 10.3389/fimmu.2014.00466 25324843 PMC4179512

[22] Li Y, Wang X, Blau DM, et al. Global, regional, and national disease burden estimates of acute lower respiratory infections due to respiratory syncytial virus in children younger than 5 years in 2019: a systematic analysis. *Lancet.* 2022;399(10340):2047-2064. 10.1016/s0140-6736(22)00478-0 35598608 PMC7613574

[23] Moline HL, Toepfer AP, Tannis A, et al. Respiratory syncytial virus disease burden and nirsevimab effectiveness in young children from 2023-2024. *JAMA Pediatr*. 2025;179(2):179-187. 10.1001/jamapediatrics.2024.5572 39652359 PMC11667569

[24] Principi N, Perrone S, Esposito S. Challenges and limitations of current RSV prevention strategies in infants and young children: a narrative review. *Vaccines (Basel)*. 2025;13(7):717. 10.3390/vaccines13070717 40733694 PMC12300764

[25] Sevendal ATK, Hurley S, Bartlett AW, et al. Systematic review of the efficacy and safety of RSV-specific monoclonal antibodies and antivirals in development. *Rev Med Virol*. 2024;34(5):e2576. 10.1002/rmv.2576 39209729

