# Supplementary material for: Optimizing the sensitivity of detection of respiratory syncytial virus infections in longitudinal studies using the combination of weekly sample testing and biannual serology
Source: Am J Epidemiol. 2025 Dec 9;195(6):1605–14. doi: 10.1093/aje/kwaf271 (PMC12767786; doi:10.1093/aje/kwaf271)
Supplement: Web_Material_kwaf271 [file web_material_kwaf271.zip › supplemental_tables.docx]

Optimizing the sensitivity of detection of respiratory syncytial virus infections in longitudinal studies using the combination of weekly sample testing and biannual serology

Shannon C. Conrey^1,2^, Daniel C. Payne^3,4^, Maria Deza Leon^5^, Monica Epperson^6^, Melissa M. Coughlin^6^, Allison R. Burrell^3^, Claire P. Mattison^6,7^, Rachel M. Burke^8^, Julia M. Baker^6^, Natalie J. Thornburg^6^, Meredith L. McMorrow^6^, Mary Allen Staat^3,4^, Ardythe L. Morrow^2,4^

^1^ Case Western Reserve University School of Medicine, Department of Quantitative and Population Health Sciences, Cleveland, OH

^2^ University of Cincinnati College of Medicine, Department of Environmental and Public Health Sciences, Cincinnati, OH

^3^ Cincinnati Children’s Hospital Medical Center, Division of Infectious Disease, Cincinnati, OH

^4^ University of Cincinnati College of Medicine, Department of Pediatrics, Cincinnati, OH

^5^ Children’s Mercy Kansas City, Department of Infectious Disease, Kansas City, MO

^6^ Centers for Disease Control and Prevention, National Center for Immunization and Respiratory Diseases, Atlanta, GA

^7^ Cherokee Nation Operational Solutions, Tulsa, OK

^8^ The Gates Foundation, Seattle, WA

**Corresponding author:**

Shannon Conrey, [shannon.conrey@case.edu](mailto:shannon.conrey@case.edu)

Included materials

Table S1: Agreement between methods used to identify RSV infections in 53 highly-adherent PREVAIL children

Table S2: Comparison between concentration change method and ROC-derived seropositivity in 194 evaluable children in the PREVAIL Cohort

Table S1: Agreement between methods used to identify RSV infections in 53 highly-adherent PREVAIL children

|  |  | Concentration change  log_10_ AU  IgA >0.202 or IgG>0.32 | | | Fold change in concentration  IgA>1.594X or IgG>2.089X | | | Concentration log_10_ AU  IgA >2.704 or  IgG>4.035 | | | 4-fold change in concentration | | |
| --- | --- | --- | --- | --- | --- | --- | --- | --- | --- | --- | --- | --- | --- |
|  |  | Pos | Not  Pos | %  agree | Pos | Not  Pos | %  agree | Pos | Not  Pos | %  agree | Pos | Not  Pos | %  agree |
| Number of children seropositive for RSV | | | | | | | | | | | | | |
| ROC-derived IgA^1^ | Pos | 34 | 1 | 94.3% | 34 | 1 | 92.4% | 35 | 0 | 88.7% | 33 | 2 | 94.3% |
|  | Not Pos | 2^2^ | 16 |  | 3^2^ | 15 |  | 6 | 12 |  | 1 | 17 |  |
| Number of incident infections | | | | | | | | | | | | | |
| RT-qPCR | Pos | 42 | 7 | 88.9% | 42 | 7 | 89.3% | 46 | 3 | 48.1% | 39 | 10 | 89.6% |
|  | Not Pos | 25 | 215 |  | 24 | 216 |  | 147 | 93 |  | 20 | 220 |  |
| Concentration change  log_10_ AU  IgA >0.202 or IgG>0.32 | Pos |  |  |  | 66 | 1 | 99.7% | 67 | 0 | 56.4% | 59 | 8 | 97.2% |
|  | Not Pos |  |  |  | 0 | 222 |  | 126 | 96 |  | 0 | 222 |  |
| Fold change in concentration  IgA>1.594X or IgG>2.089X | Pos |  |  |  |  |  |  | 66 | 0 | 56.1% | 59 | 7 | 97.6% |
|  | Not Pos |  |  |  |  |  |  | 127 | 96 |  | 0 | 223 |  |
| Concentration log_10_ AU  IgA >2.704 or  IgG>4.035 | Pos |  |  |  |  |  |  |  |  |  | 59 | 134 | 53.6% |
|  | Not Pos |  |  |  |  |  |  |  |  |  | 0 | 96 |  |

Mixed effects classification and regression tree (CART) analysis was used to identify thresholds of positivity using pre-fusion F IgA and IgG assays from serum collected at 6 weeks and 6, 12, 18, and 24 months of age. Weekly, participants submitted a nasal swab tested for respiratory syncytial virus (RSV) using a real-time polymerase chain reaction (RT-qPCR). Participants were included in the analysis if they submitted ≥90% of weekly samples (n=53). Children with a RT-qPCR positive in the interval between blood draws were identified as positive during the interval. Four variables were considered for partitioning the data; Fold change in concentration, change in log_10_ concentration, log_10_ concentration, and four-fold change in concentration. Each model was run independently for IgA and IgG. Models were run first as univariable models to establish thresholds in each variable, then as a full model to select the thresholds that produced the most accurate prediction. No limit was assigned to the number of splits. CART selected a >2.02 change in log_10_ concentration of IgA or a >0.32 change in log_10_ concentration of IgG as the most predictive threshold for use in identifying RSV infections.

^1^Seropositivity by the laboratory-determined ROC curves was limited to IgA, as 100% of children were seropositive by IgG from birth until age 6 months. Values represent the number of children positive for RSV, not the number of infections.

^2^Positive in the ROC analysis by IgG

^3^Values represent the number of infections detected using each method.

Table S2: Comparison between concentration change method and ROC-derived seropositivity in 194 evaluable children in the PREVAIL Cohort

|  |  | Concentration change  log_10_ AU  IgA >0.202 or IgG>0.32 | |  |
| --- | --- | --- | --- | --- |
|  |  | Pos | Not Pos | % agreement |
| ROC-derived IgA seropositivity | Pos | 140 | 0 | 98.5% |
|  | Not pos | 3* | 51 |  |
| Legend:  Mixed effects classification and regression tree (CART) analysis was used to identify thresholds of positivity of >2.02 change in log_10_ concentration of IgA or a >0.32 change in log_10_ concentration of IgG as the most predictive threshold for use in identifying RSV infections. Seropositivity by the laboratory-determined ROC curves was limited to IgA, as 100% of children were seropositive by IgG from birth until age 6 months   - The three participants identified as seropositive using the CART-defined thresholds were positive by IgG only and confirmed as positive by IgG in the ROC analysis. | | | | |
